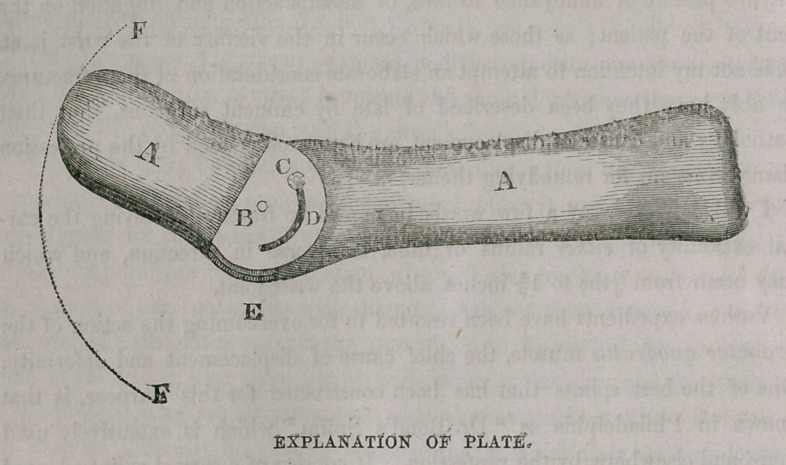# A Description of a Splint for the Treatment of Fracture of the the Carpal Extremity of the Radius or Ulna

**Published:** 1853-09

**Authors:** Ellery P. Smith

**Affiliations:** Buffalo, N. Y.


ART. V.—*A description of a Splint for the treatment of Fracture of the the Carpal Extremity of the Radius or Ulna.* Invented by ELLERY P. SMITH, M. D., of Buffalo, N. Y.

Of all fractures that the surgeon is called upon to treat, there are few, if any, so prolific of annoyance to him, of dissatisfaction and litigation on the part of the patient; as those which occur in the vicinity of the wrist joint. It is not my intention to attempt an elaborate consideration of these fractures, so ably have they been described of late by eminent surgeons, that their pathology and causes of displacement, are better understood by the profession than the means for remedying them.

I will, however, say a few words in regard to fracture involving the carpal extremity of *either* radius or ulna, transverse in direction, and which may occur from $\frac{3}{4}$ths to $1\frac{1}{2}$ inches above the wrist joint.

Various expedients have been resorted to for overcoming the action of the *pronator quadratus* muscle, the chief cause of displacement and deformity. One of the best splints that has been constructed for this purpose, is that known in Philadelphia as "Dr. Bond's Splint," which is extensively used there, and elsewhere, by the profession. It consists of a carved splint adapted to the inner aspect of the fore-arm, and hand, bending at an obtuse angle at its inferior extremity; there is also an oval block of wood to fill the palm of the hand. This splint fulfills well by its angle, the indication (in fracture of the radius) for bending the hand *downward*, thus overcoming the *pronator quadratus* muscle, but being immovable, or rigid, its angle cannot be adapted to the exigencies of different cases of fracture. Again, in fracture of the ulna, at the same distance from the wrist, a splint, with the angle reversed, to tilt the hand *up*, must be employed; and moreover, a complete set for *each hand* must be in possession of the surgeon.

It was with a view to remove, if possible, these difficulties, that I have invented a splint that embraces in itself all that is practically useful in these cases of fracture, combining simplicity and effectiveness, which I apprehend is a great desideratum in the treatment of fractures generally. To the country practitioner, or those who possess little apparatus, it must prove of value, as by the aid of a common straight splint, or on an emergency, a strip of shingle, it may be used in treating nearly all the fractures incident to the bones of the fore-arm.

Its advantages are five-fold, and I will briefly state them:

1. It is adapted for treating fractures of the radius that may occur from three-fourths to two inches above its articulation with the carpus; or,

2. For the same fractures existing in the ulna.

3. The *angle* of the splint may be increased, or diminished, to answer the indications of different cases.

4. It can be applied with equal facility to either hand.

5. It can be used as a *straight* splint in fracture of both bones.

EXPLANATION OF PLATE.

The two splints, A, A, are halved at E, and joined by the pivot B, on which they turn in the arc of a circle. The light surface in which are the letters B, C, D, on either side, is of brass, to insure strength and stiffness. The splint is maintained at the required angle, by the nut and screw C, which pass through the slit D. The dotted lines F, F, show the arc through which the left hand splint A, may be made to move. On the side of the splint not seen in the plate, the long splint A, is hollowed out to fill the convexity of the arm, and to the short splint A, is attached an oval piece of wood to fill the palm of the hand.

## Figures and Tables

**Figure f1:**